# Endothelial EPAS1 as a prognostic and therapeutic target in acute myocardial infarction: integrative bioinformatics, Mendelian randomization and experimental validation

**DOI:** 10.3389/fphar.2026.1893164

**Published:** 2026-08-04

**Authors:** Xiaona Yang, Zhongyan Li, Lingxian Guo, Jingru Li, Huan Cheng, Xue Guan, Najie Wen, Huawei Wang, Longjun Li, Lihong Yang, Yancui Zhu, Luqiao Wang

**Affiliations:** 1 Department of Cardiology, The First Affiliated Hospital of Kunming Medical University, Kunming, Yunnan, China; 2 Department of Thyroid Surgery, Clinical Research Center for Thyyroid Disease of Yunnan Province, The First Affiliated Hospital of Kunming Medical University, Kunming, China; 3 Department of Critical Care Medicine, The First Affiliated Hospital of Kunming Medical University, Kunming, Yunnan, China

**Keywords:** acute myocardial infarction, bioinformatics, colocalization analyses, EPAS1, ferroptosis, miRNA-TF-mRNA network, phewas, summary-data-based MR

## Abstract

**Background:**

Acute myocardial infarction (AMI), characterized by acute myocardial necrosis due to coronary occlusion, is a life-threatening cardiovascular event. Ferroptosis critically contributes to ischemic injury, yet its causal regulators in AMI, particularly in endothelial cells, remain elusive.

**Methods:**

We integrated AMI transcriptomics with summary-data-based Mendelian randomization (SMR) and colocalization to identify causal ferroptosis-related transcription factor (TF). A miRNA-TF-mRNA regulatory network was constructed via miRNA-TF and TF-target prediction. Subsequent analyses, including TF binding site prediction, GSEA, GeneMANIA, gene-disease and gene-drug association screening, phenome-wide association study (PheWAS), ROC curve evaluation, and RT-qPCR validation in HUVECs, were performed to characterize the molecular mechanisms of this axis in AMI.

**Results:**

EPAS1 was identified as a causal ferroptosis-related TF in AMI (*P*_SMR_ < 0.05; *P*_HEIDI_ > 0.05). We constructed a ferroptosis-related miRNA-TF-mRNA network and identified a novel endothelial-specific axis, hsa-miR-138-5p/EPAS1/BACH1. RT-qPCR in HUVECs validated the GEO-derived expression patterns of axis components. PheWAS further revealed no significant adverse phenotypic associations for genes within this axis, supporting the druggability of approved compounds targeting it.

**Conclusion:**

This study identifies EPAS1 as a TF with suggestive genetic links to AMI risk and characterizes a novel endothelial-enriched hsa-miR-138-5p/EPAS1/BACH1 regulatory axis governing ferroptosis. This molecular cascade provides candidate molecular clues for subsequent preclinical research into cardioprotective strategies targeting ferroptosis in AMI.

## 1 Introduction

Acute myocardial infarction (AMI) remains highly prevalent and incident, despite significant advances in reperfusion therapy for its clinical management (Chong et al., 2025). Meanwhile, the occurrence of myocardial ischemia-reperfusion injury (MIRI) occurs at a substantial rate, severely limiting further improvement in therapeutic outcomes (Kaczorowski et al., 2025). In the context of accelerating global population aging, a deeper mechanistic understanding of AMI pathogenesis, uncovering novel biomarkers, and the development of more effective therapeutic strategies are critically needed to enhance early diagnosis, optimize treatment regimens, and ultimately improve patient survival.

Ferroptosis is an iron-dependent regulated cell death form resulting from excessive lipid peroxidation and loss of redox homeostasis (Zhao et al., 2025). Existing data support a crucial role for ferroptosis in both initiating and exacerbating the pathological course of AMI (Wang et al., 2025; Chen et al., 2025; Wu et al., 2024a; Wu et al., 2024b). For example, EPAS1 (endothelial PAS domain-containing protein 1) can act as a risk susceptibility factor for the occurrence of AMI by driving the ferroptosis process (Huang et al., 2024). Existing studies have confirmed that BACH1 (BTB and CNC homology 1) inhibitors can significantly alleviate myocardial ischemic injury in mice (Li et al., 2025). Therefore, targeting ferroptosis represents a promising therapeutic strategy for AMI, holding significant research value and clinical potential.

Transcription factors (TFs) constitute a group of proteins capable of sequence-specific DNA binding, thereby regulating the onset of transcription by activating or repressing target genes (Tippens et al., 2018). In contrast, MicroRNAs (miRNAs), a class of endogenous ∼22-nt non-coding RNAs, exert widespread control over cellular processes by binding to target mRNAs and downregulating gene expression through translational inhibition or accelerated decay (Corey, 2025; Bai et al., 2021; Wu et al., 2023). Mounting evidence confirms microRNAs serve vital regulatory roles in myocardial ischemic injury. miR-210 preserves cardiac function via modulating mitochondrial metabolism after infarction (Song et al., 2022), while single intravenous administration of miR-199a-5p enables long-term myocardial repair (Chen et al., 2024). TFs and miRNAs interact reciprocally to form intricate miRNA-TF-mRNA regulatory networks (Zhang et al., 2025; Li et al., 2022a; Li et al., 2022b; Li et al., 2024; Li et al., 2026). Elucidating these networks in depth is crucial for uncovering the molecular mechanisms underlying AMI and for identifying novel biomarkers and therapeutic targets.

**FIGURE 1:**
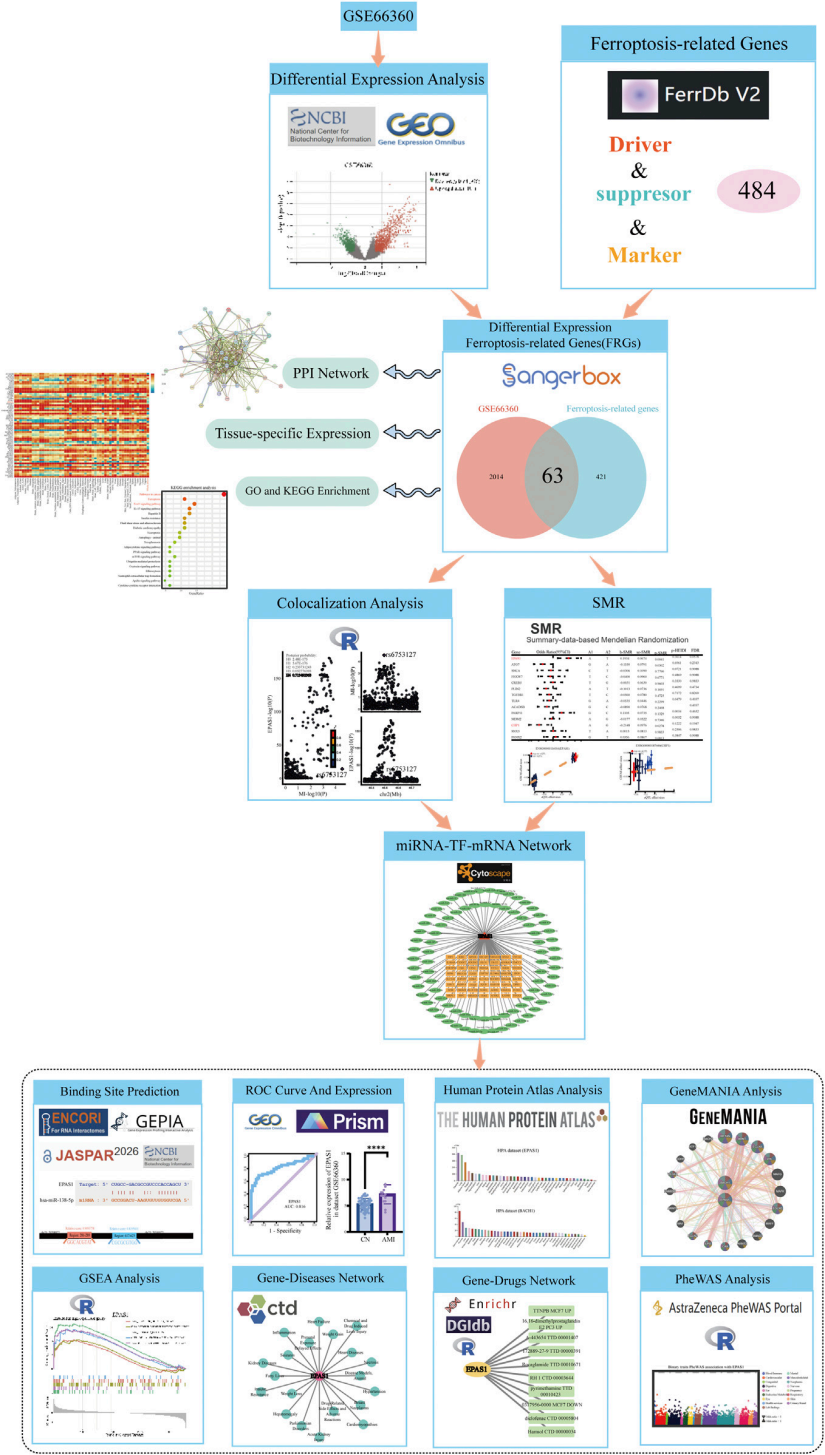
Research design flowchart of this study.

However, the precise mechanisms underlying the miRNA-TF-mRNA axis in AMI remain incompletely understood. Therefore, systematic dissection of this regulatory network holds great promise for advancing early diagnosis, prognostic stratification, and targeted therapy in AMI. Building on this rationale, this study integrates bioinformatic analyses with summary-data-based Mendelian randomization (SMR) to comprehensively identify differentially expressed miRNAs, TFs, and mRNAs in AMI, with a particular focus on their coordinated regulation of ferroptosis-related pathways. We aim to uncover novel molecular mechanisms driving AMI progression and to provide a theoretical foundation for identifying potential therapeutic targets. The pathological process of AMI is closely related to immune microenvironment imbalance and abnormal infiltration of immune cells (Jiang et al., 2025). Therefore, this study, while exploring the ferroptosis regulatory mechanism mediated by miRNA-TF-mRNA, further analyzes the characteristics of immune infiltration in AMI tissues, elucidating the pathogenesis from both molecular regulation and immune microenvironment perspectives. Figure 1 summarized and illustrated the overall workflow of this study.

## 2 Materials and methods

### 2.1 Study design

This study integrated transcriptomic profiling, genetic association data, and multi-omics regulatory resources to investigate the role of ferroptosis-related genes in AMI. Differential expression analysis of the discovery cohort was performed using the GSE66360 dataset from the Gene Expression Omnibus (GEO) to identify mRNAs dysregulated in AMI patients compared with controls, and candidate findings were externally validated in the independent validation cohort (GSE71226). These differentially expressed mRNAs were intersected with ferroptosis-related genes to define a subset of differentially expressed ferroptosis-related genes (DE-FRGs). Functional characterization of DE-FRGs was carried out through Gene Ontology (GO) and Kyoto Encyclopedia of Genes and Genomes (KEGG) enrichment analyses, protein-protein interaction (PPI) network construction, and tissue-specific expression profiling using FUMA-GENE2FUNC. To evaluate potential causal links between gene expression and AMI risk, SMR analysis was conducted by integrating cis-expression quantitative trait loci (cis-eQTL) data from the Genotype-Tissue Expression (GTEx) Project v8 (https://www.gtexportal.org/) (Barbeira et al., 2021) with AMI genome-wide association study (GWAS) summary statistics from the IEU OpenGWAS database; this was followed by heterogeneity in dependent instruments (HEIDI) testing and Bayesian colocalization analysis. Upstream miRNAs targeting hub gene were predicted by overlapping results from ENCORI, TargetScan, and miRWalk databases, while downstream targets were identified using ChIP-seq data from ChIP-Atlas and Cistrome DB. Candidate regulatory axes were further refined through differential expression screening in an independent AMI cohort (GSE148153) alongside GSE66360. Finally, the biological relevance, diagnostic potential, and therapeutic implications of the prioritized axis were evaluated via transcription factor binding site prediction, gene set enrichment analysis (GSEA), GeneMANIA interaction mapping, phenome-wide association study (PheWAS), and receiver operating characteristic (ROC) curve analysis and RT-qPCR validation.

### 2.2 Data sources

Transcriptomics data were sourced from the GEO database (https://www.ncbi.nlm.nih.gov/geo/) (Barrett et al., 2013). The mRNA expression profiles used for differential expression analysis were derived from dataset GSE66360 (Muse et al., 2017), which includes 50 control samples and 49 AMI samples. For auxiliary reference of hub gene expression trends, we additionally analyzed transcriptomic profiles from GSE71226. For miRNA differential expression analysis, dataset GSE148153 (Nie et al., 2020) was utilized, comprising 7 control samples and 5 AMI samples. Detailed information is provided in Table 1. 484 ferroptosis-related genes were retrieved from the FerrDb database (http://www.zhounan.org/ferrdb/current/) (Zhou N. et al., 2023), including established markers, drivers, and suppressors of ferroptosis (Supplementary Table S1). Additionally, 68 ferroptosis-related miRNAs (Supplementary Table S2) were obtained from the ncFO database (http://www.jianglab.cn/ncFO/) (Zhou S. et al., 2023).

**TABLE 1:** Details of the relevant gene expression profiles applied in this study.

Dataset	Platform	Control	AMI	Country	Submission	Samples	Application
GSE66360	GPL570	50	49	United States of America	2015	Circulating endothelial cells	Identification and validation for DE-mRNAs
GSE71226	GPL570	3	3	China	2015	Peripheral blood	Auxiliary reference of hub gene expression trends
GSE148153	GPL20712	7	5	China	2020	Peripheral blood serum	Identification for DE-miRNAs

All transcriptomic data of GSE66360 were generated from CD146-positive circulating endothelial cells (CECs) enriched from peripheral blood, instead of unfractionated whole blood bulk RNA. GSE71226 contains patients with general coronary heart disease (CHD) without exclusive acute myocardial infarction (AMI) subgroups, thus only used as an auxiliary expression reference rather than an independent AMI, validation cohort.

### 2.3 Acquisition of DE-FRGs

Differentially expressed mRNAs (DE-mRNAs) and miRNAs (DE-miRNAs) between AMI and control samples were identified using GEO2R (https://www.ncbi.nlm.nih.gov/geo/geo2r/), an online tool based on the limma R package. GEO2R was used with all analytical parameters kept at default settings. Briefly, raw expression data were processed by quantile normalization and log_2_ transformation. The limma package was applied to assess differential expression, and the Benjamini–Hochberg false discovery rate (FDR) was adopted for multiple testing correction. Differentially expressed genes and miRNAs were identified using these thresholds: adjusted P < 0.05 and |log_2_FC| > 0.585. Subsequently, differentially expressed ferroptosis-related genes (DE-FRGs) and ferroptosis-related miRNAs (DE-FRmiRNAs) were obtained by intersecting the DE-mRNAs with ferroptosis-related genes (FRGs) and the DE-miRNAs with known ferroptosis-associated miRNAs, respectively.

### 2.4 GO and KEGG enrichment analyses

GO and KEGG pathway enrichment analyses of the DE-FRGs were performed as previously described (Li et al., 2024). Significantly enriched terms and pathways were defined as those with an P-value <0.05.

### 2.5 PPI network construction

A network of protein interactions among DE-FRGs was developed through the STRING database (https://cn.string-db.org/) with *Homo sapiens* as the reference species. Only interactions with a minimum required confidence score of 0.4 (medium confidence) were included, and disconnected nodes were hidden.

### 2.6 Heatmap and tissue-specific expression profiling of DE-FRGs

Based on the GSE66360 dataset, DE-FRGs were selected for heatmap visualization to compare their expression profiles between the control and AMI groups. To further characterize their tissue-specific expression patterns, we performed gene expression enrichment analysis across 54 human tissues using the FUMA GENE2FUNC platform (https://fuma.ctglab.nl/gene2func) (Watana et al., 2017), which leverages transcriptomics data from the GTEx project.

### 2.7 SMR analysis

To investigate potential causal relationships between DE-FRGs and AMI, we performed SMR analysis using the SMR software (https://cnsgenomics.com/software/smr/) (Zhu et al., 2016). This approach integrates GWAS and eQTL summary data to test for causal associations between gene expression and traits, distinguishing causal effects from linkage disequilibrium confounding to identify potential causal genes underlying disease risk (Wu et al., 2018). For the exposure data, we utilized cis-eQTL summary statistics derived from whole blood tissue in the GTEx Project. For the outcome, we obtained GWAS summary statistics for AMI from the IEU OpenGWAS database (https://gwas.mrcieu.ac.uk/) under study accession GCST90018877 (genome build: GRCh37/hg19) (Sakaue et al., 2021).

Key parameter settings for the SMR analysis were as follows. The cis-region for each gene was defined as ±1 Mb around its transcription start site (TSS), consistent with standard practice in eQTL-based Mendelian randomization studies (Larsson et al., 2023). For each gene, the lead cis-eQTL was selected as the instrumental variable. To ensure high-confidence instruments, only genes whose lead cis-eQTL achieved a significance level of *P*_SMR_ < 5 × 10^−8^ in the exposure dataset were included in downstream SMR testing. Additional default settings in the SMR software were applied: HEIDI heterogeneity test was performed using a default window size of 1,000 kb and an LD *r* ^2^ threshold of 0.9 for clumping proximal variants (Cheng et al., 2025).

The HEIDI test (*P*_HEIDI_ > 0.05) excluded genes with multiple linked causal variants. Genes satisfying nominal raw (*P*_SMR_ < 0.05) together with (*P*_HEIDI_ > 0.05) were defined as suggestive AMI causal candidate genes for downstream analyses. The Benjamini–Hochberg FDR correction was subsequently performed across all 63 tested DE-FRGs in R to evaluate multiple testing bias, yet FDR-adjusted P value was not adopted as a filtering threshold for candidate identification.

### 2.8 Bayesian colocalization analysis

To test for shared causal variants between gene expression of the DE-FRGs and AMI, we performed Bayesian colocalization analysis using the coloc R package (Seefelder et al., 2024). For each of the DE-FRGs, we used the same cis-eQTL (GTEx v8, whole blood) and AMI GWAS (IEU OpenGWAS, GCST90018877) summary statistics as described in the SMR analysis above. As described in previous literature, Bayesian colocalization analysis involves five posterior probability hypotheses (Cai et al., 2023). Genes with a posterior probability for H_4_ (PPH4) ≥ 0.7 were considered to have strong evidence of colocalization (Wallace, 2021; Zou M. et al., 2024), indicating that the same underlying genetic variant likely influences both gene expression and AMI risk.

### 2.9 Construction of miRNA-TF-mRNA regulatory network

To Illuminate the transcriptional and post-transcriptional regulatory mechanisms underlying AMI, we constructed a miRNA-TF-mRNA network centered on a key ferroptosis-related TF identified in our prior analyses.

First, upstream miRNAs potentially targeting the TF were predicted using three complementary databases: ENCORI (https://rnasysu.com/encori/) (Li et al., 2014), TargetScan (https://www.targetscan.org/vert_80/) (McGeary et al., 2019), and miRWalk (http://mirwalk.umm.uni-heidelberg.de/) (Sticht et al., 2018). Only miRNAs consistently predicted by three platforms were retained to enhance prediction reliability. Second, downstream target genes of the TF were retrieved from two ChIP-seq data resources: ChIP-Atlas (https://chip-atlas.org/) (Zou Z. et al., 2024) and Cistrome DB (http://cistrome.org/) (Zheng et al., 2019), focusing on high-confidence binding peaks in relevant cardiovascular or immune cell types. Third, to narrow the candidate regulators, we intersected the predicted upstream miRNAs with differentially expressed miRNAs from GSE148153, and the predicted TF target genes with differentially expressed mRNAs from GSE66360 (|log_2_FC| > 0.585, adjusted P < 0.05).

By combining computational predictions with actual expression changes observed in AMI, these approach identified miRNA-TF-mRNA interactions most likely involved in ferroptosis. Among these, a core regulatory axis consisting of miRNA-TF-mRNA was ultimately retained as the candidate ferroptosis-related pathway in AMI.

### 2.10 Binding site prediction

To characterize the molecular interactions within the identified miRNA-TF-mRNA axis, we predicted binding sites at both regulatory levels. Firstly, the putative binding sites of the candidate miRNA on the 3′ untranslated region (3′UTR) of the TF transcript were retrieved from the ENCORI database. Secondly, transcription factor binding sites (TFBSs) in the promoter regions of downstream target mRNAs were predicted using the JASPAR database (https://jaspar.genereg.net/) (Ovek Baydar et al., 2026), a curated database of TF binding profiles based on position weight matrices, with a relative profile score threshold >80%. Finally, we evaluated the expression correlation between the TF and its target mRNA in whole blood using the GEPIA2 platform (http://gepia2.cancer-pku.cn/), based on GTEx RNA-seq data and Pearson’s correlation coefficient (P < 0.05 considered significant).

### 2.11 Evaluation of diagnostic potential and RT-qPCR validation of key genes

We assessed the diagnostic value of key genes using ROC curve analysis in the GSE66360 dataset. Area under the curve (AUC) values were calculated to evaluate discriminatory power between AMI patients and controls (Nahm, 2022). An AUC value >0.8 is generally considered to have good diagnostic utility (Çorbacıoğlu and Aksel, 2023). Gene expression levels in AMI versus control samples from the same dataset were visualized using bar plots. Protein-level expression across human tissues was retrieved from The Human Protein Atlas (https://www.proteinatlas.org/).

Human umbilical vein endothelial cells (HUVECs) were purchased from Fenghui Biotechnology (Changsha, China) and maintained at 37 °C under 5% CO_2_ in endothelial cell medium containing 10% fetal bovine serum. To model AMI *in vitro*, cells were subjected to oxygen-glucose deprivation (OGD) by replacing the medium with glucose-free, serum-free DMEM and incubating them in a modular hypoxia chamber flushed with 95% N_2_/5% CO_2_ for 4 h at 37 °C.

To validate the key genes, reverse transcription-quantitative polymerase chain reaction (RT-qPCR) was performed on HUVECs. Total RNA was extracted using the TransZol Up Plus RNA Kit, and cDNA synthesis and qPCR was carried out using the TransScript® Uni Cells to CT 1-Step Probe Kit. Relative gene expression was calculated using the 2^−ΔΔCT^ method with β-actin as the endogenous reference gene. Given the small sample size (n = 3 independent biological replicates per group) and to avoid the assumption of equal variances, statistical comparisons between normoxic control and OGD-treated groups were performed using an unpaired two-tailed t-test with Welch’s correction implemented in GraphPad Prism (version 10). And statistical significance was set at *P* < 0.05. All bar charts display data as mean ± standard deviation (SD) calculated from three independent biological replicates. RT-qPCR was performed using the following primers: EPAS1 forward 5′-TAG​CCC​TGT​CCA​ACA​AGC​TG-3′ and reverse 5′-CAA​GTG​TGA​ACT​GCT​GGT​GC-3'; BACH1 forward 5′-CAC​AAA​GTG​CAA​AGA​CCC​CG-3′ and reverse 5′-ATC​GCC​TGA​CTG​CTC​GTA​TG-3'; β-actin forward 5′-TGC​TAT​GTT​GCC​CTA​GAC​TTC​G-3′ and reverse 5′-GTT​GGC​ATA​GAG​GTC​TTT​ACG​G-3'.

### 2.12 GeneMANIA/GSEA analysis

We performed GeneMANIA (http://genemania.org/) (Franz et al., 2018) to construct a functional association network for the key genes, integrating evidence from physical and genetic interactions, co-expression, co-localization, and shared pathway annotations. In this study, we explored the top 20 functionally similar genes of hub genes and performed functional enrichment analysis. We performed gene set enrichment analysis (GSEA) for each core gene using the clusterProfiler R package (Canzler and Hackermüller, 2020) and KEGG gene sets from MSigDB (C2 collection) were used as reference. Enriched pathways were defined as those with an adjusted P-value <0.05. Results were visualized as ridge plots based on normalized enrichment score (NES) using the enrichplot package.

### 2.13 Gene-disease and gene–drug association analysis

We explored disease associations of the key genes using the Comparative Toxicogenomics Database (CTD; http://ctdbase.org/) (Davis et al., 2023), which curates experimentally supported gene-disease relationships. For each gene, the top 20 diseases with the highest inference scores were retained for network visualization. Gene-drug interactions were retrieved from the MISGDB database (http://www.misgdb.org/) (Castanza et al., 2023), a resource integrating multi-omics evidence for drug-gene links. The top 10 drugs with the strongest association scores for each key gene were selected.

### 2.14 Phenome-wide association analysis

We used the AstraZeneca PheWAS Portal (https://azphewas.com/) (Wang et al., 2021; Dhindsa et al., 2023) to systematically evaluate associations between candidate genes and a wide range of human phenotypes. This platform enables systematic investigation of phenotypic traits associated with candidate genes, facilitating the identification of adverse effects and potential safety liabilities in a drug development context.

## 3 Results

### 3.1 Identification of DE-FRGs

**FIGURE 2:**
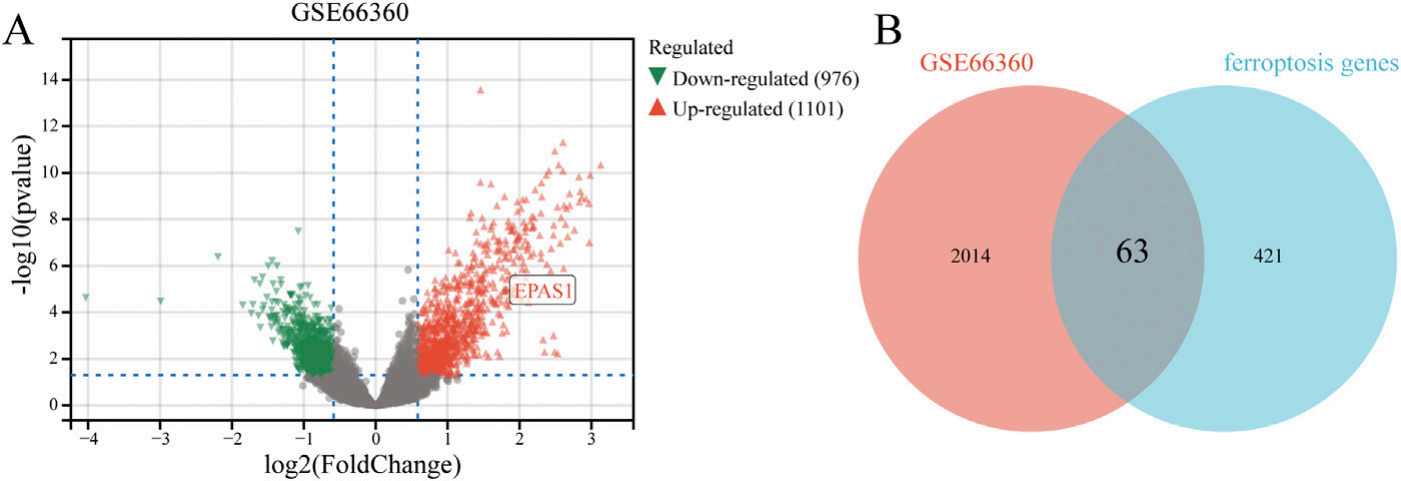
Differential analysis of GSE66360. **(A)** Volcano plot displays the expression profile of 2,077 DE-mRNAs in the GSE66360 dataset. Red triangles represent upregulated genes, gray dots represent non-significant genes, and green triangles represent downregulated genes. **(B)** Venn diagram illustrates the intersection between 2,077 DE-mRNAs and 484 ferroptosis-related genes. The pink circle represents the DE-mRNAs from the GSE66360 dataset, while the blue circle signifies the ferroptosis-related genes. As depicted in the diagram, there are 63 DE-FRGs.

In the GSE66360 dataset, 2,077 differentially expressed genes (DEGs) were identified between AMI and healthy control groups, of which 1,101 were upregulated and 976 were downregulated in AMI patients (Figure 2A). By intersecting these DEGs with a curated set of 484 FRGs, we obtained 63 DE-FRGs (Figure 2B), which serve as key candidates for further investigation into the role of ferroptosis in AMI.

### 3.2 GO and KEGG pathway analyses of DE-FRGs

**FIGURE 3:**
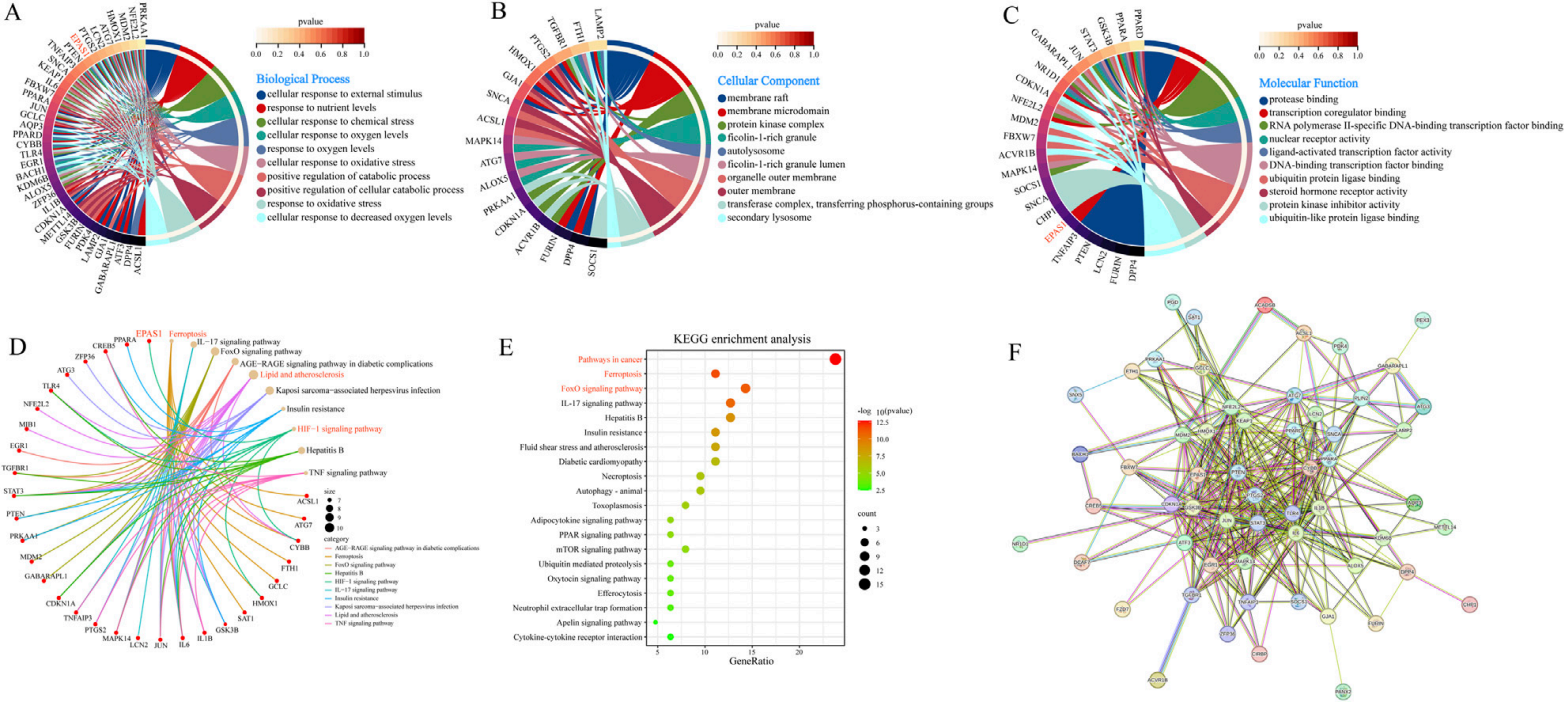
GO and KEGG pathway analyses and PPI network of DE-FRGs. **(A)** GO biological process (BP) enrichment chord diagram of DE-FRGs. Genes are displayed on the left, and enriched GO-BP terms on the right. The connecting lines show the involvement of each gene in the corresponding biological processes. The right outer color gradient reflects enrichment significance based on P value. **(B)** GO cellular component (CC) enrichment chord diagram of DE-FRGs. **(C)** GO molecular function (MF) enrichment chord diagram of DE-FRGs. **(D)** Chord diagram illustrating KEGG pathway enrichment of DE-FRGs. Red nodes represent individual DE-FRGs, and yellow nodes represent significantly enriched KEGG pathways. Node size is proportional to the number of genes associated with each pathway. **(E)** Bubble diagram illustrates the significant enriched KEGG pathway analysis of the 63 DE-FRGs. Node size is proportional to the number of genes associated with each pathway. Color scale: −log_10_(P value). Red indicates smaller P values (higher significance); green indicates larger P values (lower significance). **(F)** PPI networks of 63 DE-FRGs (confidence score ≥0.4). Nodes stand for proteins, and edges indicate interaction relationships.

To elucidate the biological roles of the 63 DE-FRGs, we performed GO enrichment analysis. In the biological process (BP) category, DE-FRGs were overwhelmingly enriched in stress-metabolism-responsive pathways, including regulation of cellular response to external stimulus (P < 0.001), response to nutrient levels (P < 0.001), and cellular response to chemical stress (P < 0.001) (Figure 3A). In the cellular component (CC) category, DE-FRGs were significantly localized to specialized membrane microdomains and signaling complexes, particularly membrane rafts (P < 0.001), membrane microdomains (P < 0.001), and protein kinase complexes (P < 0.001) (Figure 3B). In the molecular function (MF) category, DE-FRGs showed strong enrichment in regulatory protein-protein interactions, notably protease binding, transcription coregulator binding, and RNA polymerase II-specific DNA-binding transcription factor binding (P < 0.001) (Figure 3C). Together, these results indicate that DE-FRGs are functionally integrated into stress-sensing, membrane-associated signaling hubs and transcriptional regulatory networks that may orchestrate ferroptosis in the context of AMI.

KEGG pathway analysis of the 63 DE-FRGs identified 10 significantly enriched pathways (all P < 0.05), with the top hits strongly implicating regulated cell death, inflammatory signaling, and hypoxia-related signaling pathways (Figures 3D,E). Most notably, ferroptosis emerged as the second most significantly enriched pathway (P = 3.02 × 10^−12^), directly validating our focus on iron-dependent cell death.

A PPI network was constructed for the 63 DE-FRGs using the STRING database, retaining only interactions with a confidence score >0.4 to ensure medium reliability. The resulting network included 63 nodes and 336 edges (Figure 3F). Notably, EPAS1, a key hypoxia-responsive transcription factor under investigation (Pirri et al., 2024), emerged as a central node within this network, suggesting its potential role in coordinating ferroptosis-related signaling in AMI.

### 3.3 Heatmap and tissue-specific expression profiling of the DE-FRGs

**FIGURE 4:**
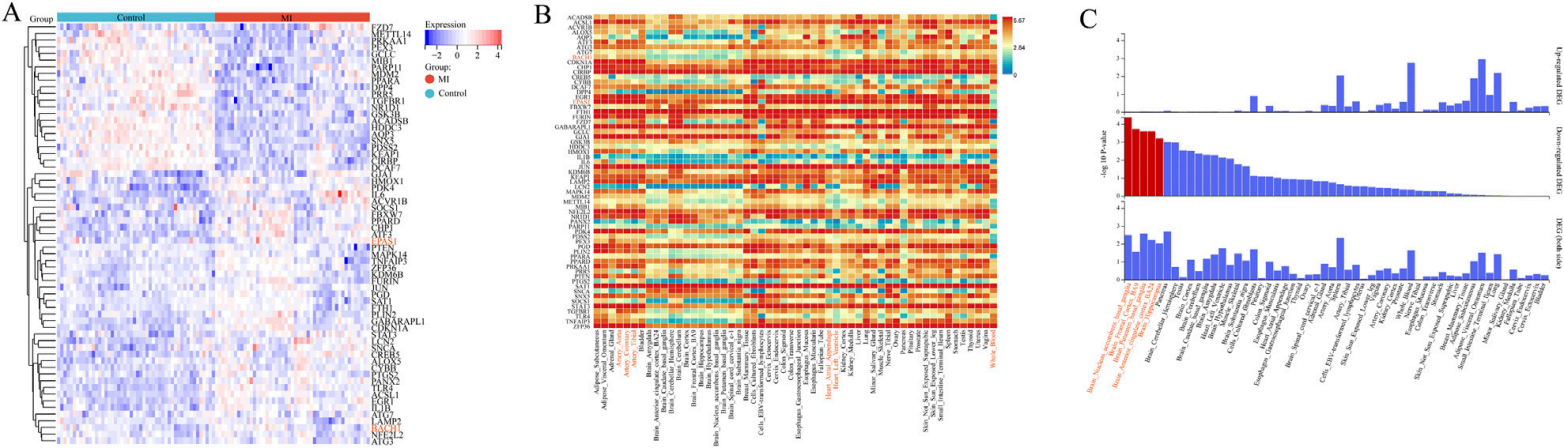
Gene-expression heatmap and tissue-specific expression pattern. **(A)** Expression heatmap for top 63 DE-FRGs. Color gradient represents the relative expression level; red indicates high expression, while blue indicates low expression. **(B)** Heatmap demonstrates tissue-specific expression patterns of 63 DE-FRGs. Analysis was conducted using GENE2FUNC in FUMA, based on the GTEx v8 database comprising 54 tissue types. The x-axis represents different tissues, while the y-axis lists the genes analyzed. The color gradient corresponds to the expression levels, with blue indicating low expression, yellow indicating moderate expression, and red representing high expression. **(C)** Plot shows the differential expression of 63 genes in different tissues.

The heatmap in Figure 4A displays the expression profiles of 63 DE-FRGs across control and AMI samples. Detailed information on these 63 DE-FRGs was provided in Table 2. Clustering based on these genes distinctly separated the AMI and control groups, demonstrating their potential as discriminative biomarkers. Notably, EPAS1 and BACH1 were significantly upregulated in AMI samples compared to controls.

**TABLE 2:** Detailed information of 63 DE-FRGs.

Genes	Full name	Role in ferroptosis	logFC	P.Value	adj.P.Val
ACADSB	Acyl-CoA dehydrogenase short/branched chain	Driver	−0.8136	0.0001	0.0042
ACSL1	Acyl-CoA synthetase long-chain family member 1	Driver	2.0929	0.0000	0.0000
ACVR1B	Activin A receptor, type IB	Driver	0.6424	0.0001	0.0044
ALOX5	Arachidonate 5-Lipoxygenase	Driver	0.7358	0.0012	0.0235
AQP3	Aquaporin 3	Driver	−0.6641	0.0002	0.0059
ATF3	Activating transcription factor 3	Driver	1.4815	0.0000	0.0000
ATG3	Autophagy related 3	Driver	0.8031	0.0003	0.0074
ATG7	Autophagy related 7	Driver	1.1065	0.0003	0.0082
BACH1	BTB and CNC homology 1	Driver	1.4611	0.0000	0.0000
CDKN1A	Cyclin-dependent kinase inhibitor 1A	Suppresor	1.6906	0.0000	0.0000
CHP1	Calcineurin-like EF-hand protein 1	Driver	1.1405	0.0000	0.0000
CIRBP	Cold inducible RNA binding protein gene	Driver	−1.0383	0.0005	0.0117
CREB5	CAMP responsive element binding protein 5	Suppresor	1.0787	0.0001	0.0023
CYBB	Cytochrome b-245 beta chain	Driver	0.9401	0.0001	0.0050
DCAF7	DDB1- and CUL4-associated factor 7	Driver	−0.9278	0.0000	0.0020
DPP4	Dipeptidyl Peptidase-4 gene	Driver	−0.7459	0.0003	0.0092
EGR1	Early growth response protein 1 gene	Driver	1.6251	0.0000	0.0003
EPAS1	Endothelial PAS domain protein 1 gene	Driver	1.8642	0.0000	0.0000
FBXW7	F-box and WD repeat domain containing 7 gene	Driver	0.8951	0.0001	0.0030
FTH1	Ferritin heavy polypeptide 1	Driver	1.7818	0.0000	0.0000
FURIN	Furin gene	Suppresor	0.9703	0.0001	0.0028
FZD7	Frizzled class receptor 7	Suppresor	−0.8068	0.0006	0.0142
GABARAPL1	Gamma-aminobutyric acid receptor-associated protein-like 1 gene	Driver	1.4584	0.0000	0.0000
GCLC	Glutamate-cysteine ligase, catalytic subunit gene	Suppresor	−0.7148	0.0022	0.0351
GJA1	Gap junction protein, alpha 1	Driver	1.5308	0.0002	0.0057
GSK3B	Glycogen synthase kinase 3 beta	Driver	−0.7911	0.0010	0.0197
HDDC3	HD domain containing 3	Driver	−0.6698	0.0001	0.0046
HMOX1	Heme oxygenase (decycling) 1	Driver	0.9140	0.0001	0.0050
IL1B	Interleukin 1 beta gene	Driver	2.8660	0.0000	0.0000
IL6	Interleukin 6 gene	Driver	0.6003	0.0003	0.0078
JUN	Jun proto-oncogene	Suppresor	1.8418	0.0000	0.0000
KDM6B	Lysine demethylase 6B gene	Driver	0.9243	0.0000	0.0022
KEAP1	Kelch-like ECH-associated protein 1	Driver	−0.6368	0.0025	0.0383
LAMP2	Lysosomal-associated membrane protein 2	Suppresor	0.8399	0.0022	0.0356
LCN2	Lipocalin 2	Suppresor	0.8592	0.0012	0.0230
MAPK14	Mitogen-activated protein kinase 14	Driver	0.6300	0.0017	0.0290
MDM2	Murine double minute 2	Driver	−0.9117	0.0000	0.0017
METTL14	Methyltransferase-like 14	Driver	−0.8260	0.0003	0.0091
MIB1	Mindbomb E3 ubiquitin protein ligase 1	Driver	−0.6207	0.0034	0.0472
NFE2L2	Nuclear factor, erythroid 2 like 2	Driver	0.6857	0.0001	0.0025
NR1D1	Nuclear receptor subfamily 1, group D, member 1	Driver	−0.7238	0.0005	0.0130
PANX2	Pannexin 2	Suppresor	1.1360	0.0000	0.0000
PARP11	Poly (ADP-ribose) polymerase family, member 11	Suppresor	−0.9366	0.0000	0.0004
PDK4	Pyruvate dehydrogenase kinase 4	Suppresor	2.1251	0.0000	0.0000
PDSS2	Prenyl (decaprenyl) diphosphate synthase, subunit 2	Suppresor	−0.8283	0.0023	0.0357
PEX3	Peroxisomal biogenesis factor 3	Driver	−0.7798	0.0004	0.0103
PGD	Preimplantation genetic diagnosis	Driver	0.9901	0.0000	0.0003
PLIN2	Perilipin 2	Suppresor	0.9808	0.0000	0.0000
PPARA	Peroxisome proliferator-activated receptor alpha	Suppresor	−0.7549	0.0000	0.0005
PPARD	Peroxisome proliferator-activated receptor alpha	Suppresor	1.0052	0.0000	0.0003
PRKAA1	Protein kinase AMP-activated catalytic subunit alpha 1	Driver	−0.7143	0.0019	0.0320
PRR5	Proline rich 5,renal	Suppresor	−0.6357	0.0027	0.0402
PTEN	Gene of phosphate and tension homology deleted on chromosome ten	Driver	0.6173	0.0027	0.0404
PTGS2	Prostaglandin-endoperoxide synthase 2	Marker	1.4349	0.0002	0.0066
SAT1	Spermidine/spermine N1-acetyltransferase 1	Driver	1.2845	0.0000	0.0010
SNCA	Synuclein-alpha	Driver	0.9504	0.0002	0.0066
SNX5	Sorting nexin 5	Driver	−0.6469	0.0021	0.0343
SOCS1	Suppressor of cytokine signaling 1	Driver	1.1761	0.0000	0.0001
STAT3	Signal transducer and Activator of transcription 3	Suppresor	1.0877	0.0000	0.0001
TGFBR1	Transforming growth factor beta receptor 1	Driver	−1.0327	0.0001	0.0033
TLR4	Toll-like receptor 4	Driver	1.7911	0.0000	0.0000
TNFAIP3	Tumor necrosis factor Alpha-induced protein 3	Driver	1.5482	0.0000	0.0007
ZFP36	ZFP36 zinc finger protein	Suppresor	1.8732	0.0000	0.0000

To explore the tissue-specific expression patterns of these DE-FRGs, we performed cross-tissue expression profiling using FUMA’s GENE2FUNC module, based on GTEx v8 data. As shown in Figure 4B, the 63 DE-FRGs exhibited widespread but heterogeneous expression across various human tissues, with notable expression in multiple cardiovascular-relevant tissues, including heart (atrial and ventricular), aorta, and whole blood. Figure 4C shows the differential expression patterns of these genes across different tissues.

### 3.4 EPAS1 exhibits causal and colocalization evidence for AMI

**FIGURE 5:**
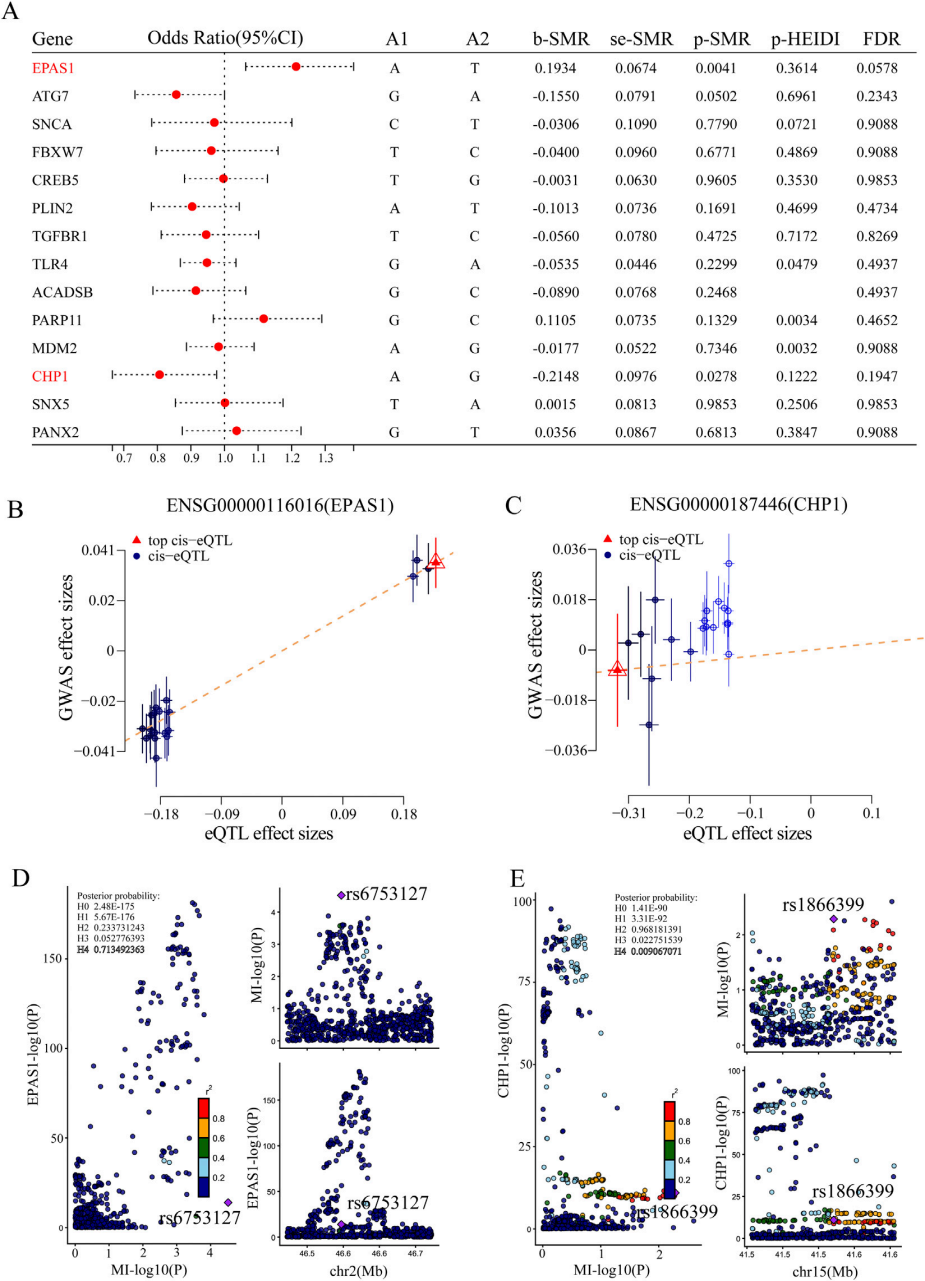
Results of SMR and colocalization analysis. **(A)** Forest plot shows significant SMR results between DE-FRGs with AMI. **(B)** Scatter plot of SMR analysis showing the association between EPAS1 gene expression and AMI. Each point represents a cis-acting eQTL instrument for the indicated gene. The horizontal axis displays the SNP effect on gene expression, and the vertical axis shows the corresponding SNP effect on AMI risk. **(C)** Scatter plot of SMR analysis showing the association between CHP1 gene expression and AMI. **(D)** Regional plot of variant-specific colocalization evidence between the EPAS1 locus and AMI. Each point represents a genetic variant. **(E)** Regional plot of variant-specific colocalization evidence between the BACH1 locus and AMI.

SMR was performed to assess the causal effects of the 63 DE-FRGs on AMI. The forest plot (Figure 5A) presents the significant results of SMR analysis, with EPAS1 and CHP1 (Calcineurin B Homologous Protein 1) showing nominal evidence of association. Specifically, higher genetically predicted expression of EPAS1 was associated with increased AMI risk (*P*_SMR_ = 0.0041, *P*_HEIDI_ = 0.3614; OR = 1.21, 95% CI: 1.06–1.38), while CHP1 showed a protective effect (*P*_SMR_ = 0.0278, *P*_HEIDI_ = 0.1222; OR = 0.81, 95% CI: 0.67–0.98). The detailed outcomes of SMR analyses can be found in Supplementary Table S3. Scatter plots of SNP-level associations for EPAS1 and BACH1 with AMI are shown in Figures 5B,C, respectively. The SNP-level scatter plot indicates a positive genetic association between EPAS1 and AMI, in agreement with the direction of effect observed in the SMR analysis.

Bayesian colocalization analysis across 806 variants spanning the EPAS1 genomic interval on chromosome 2 identified consistent evidence of a shared causal variant between EPAS1 expression and AMI risk, with the top variant rs6753127 and an overall region-wide PPH4 = 0.713 (Figure 5D), suggesting EPAS1 as a plausible mediator of genetic susceptibility to AMI. Full posterior probabilities (PP.H0–PP.H4) for this locus are reported in Supplementary Table S4. In contrast, colocalization analysis for CHP1 yielded a PPH4 < 0.5 (Figure 5E), providing no substantial evidence for a shared causal variant with AMI.

### 3.5 A ferroptosis-related hsa-miR-138-5p/EPAS1/BACH1 regulatory axis is identified in AMI

**FIGURE 6:**
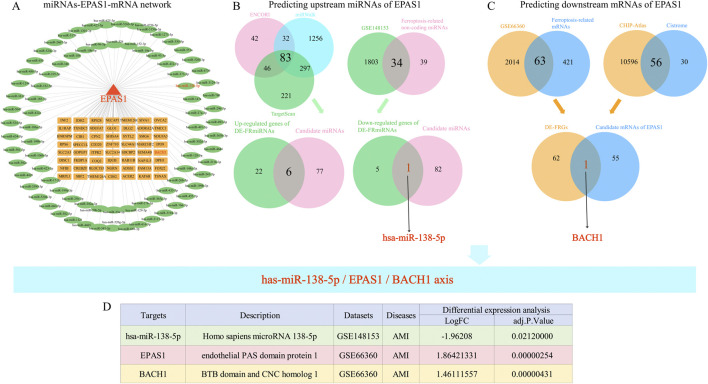
Construction of miRNA-TF-mRNA network. **(A)** Predicted miRNA-TF-mRNA regulatory network constructed using Cytoscape. Red triangles represent core transcription factors (EPAS1); green ellipses denote upstream miRNAs; orange rectangles indicate downstream target mRNAs. **(B)** Venn diagram showing the step-by-step screening process of key upstream miRNAs of EPAS1. **(C)** Venn diagram showing the step-by-step screening process of key downstream mRNAs of EPAS1. **(D)** Description of the key hsa-miR-138-5p/EPAS1/BACH1 axis designed in this article.

Figure 6A summarizes the miRNA-EPAS1-mRNA regulatory network constructed in this study. Specifically, integration of predictions from three independent online platforms identified 83 candidate miRNAs potentially targeting EPAS1. To prioritize functionally relevant miRNAs, we performed differential expression analysis using the GSE148153 dataset and intersected the results with 73 ferroptosis-related miRNAs, yielding 34 differentially expressed ferroptosis-associated miRNAs. Given the negative regulatory role of miRNAs on their target mRNAs and considering that EPAS1 was significantly upregulated in AMI patients, we focused on miRNAs that were downregulated in AMI. This led to the identification of hsa-miR-138-5p as a suitable candidate (Figure 6B).

To identify downstream effectors of EPAS1, we analyzed the GSE66360 dataset and intersected its differentially expressed genes with 484 ferroptosis-related genes, resulting in 63 overlapping genes. Concurrently, we compiled 56 putative mRNA targets of EPAS1 predicted by two independent databases. The intersection of these two gene sets revealed a single common gene, BACH1, which was also significantly upregulated in AMI samples (Figure 6C).

Collectively, these integrative analyses support the existence of a ferroptosis-associated regulatory axis in AMI: hsa-miR-138-5p/EPAS1/BACH1. Figure 6D shows the results of differential analysis of each gene in this core axis across different datasets. On this basis, we propose that in the ischemic microenvironment of AMI, downregulation of hsa-miR-138-5p alleviates its repression on the transcription factor EPAS1, leading to increased EPAS1 expression. Elevated EPAS1 subsequently drives the upregulation of BACH1, which subsequently promotes ferroptosis and thereby aggravates myocardial injury in AMI.

### 3.6 Bioinformatic evidence supports the hsa-miR-138-5p/EPAS1/BACH1 regulatory axis

**FIGURE 7:**
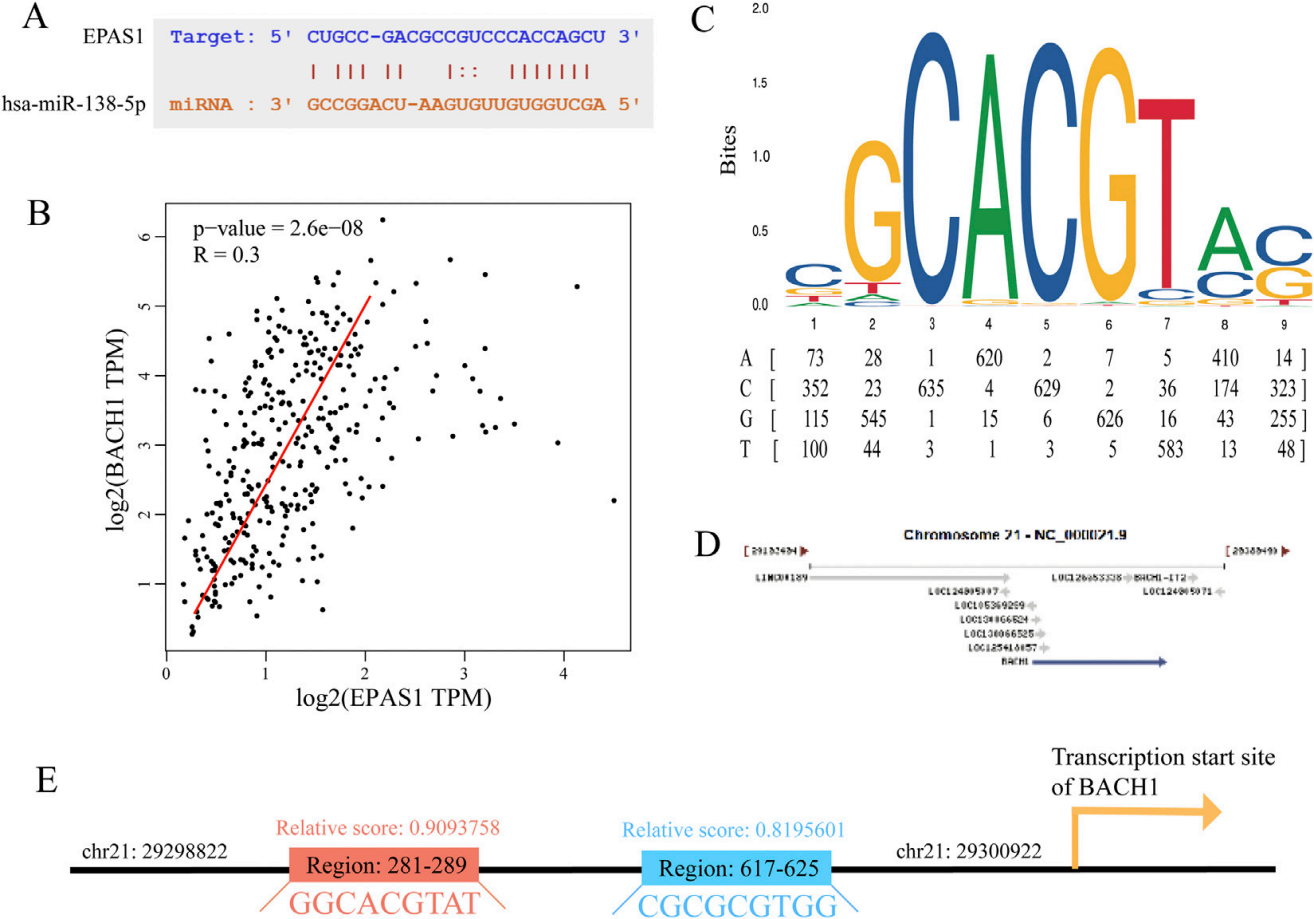
Binding site prediction of *hsa-miR-138-5p*/EPAS1/BACH1 axis. **(A)** Binding site of *hsa-miR-138-5p* and EPAS1. **(B)** Correlation analysis of EPAS1 and BACH1. **(C)** Binding motif of EPAS1 predicted by JASPAR database. **(D)** Gene transcription information of BACH1. **(E)** Predicted binding site of EPAS1 and BACH1.

To explore the molecular basis of the hsa-miR-138-5p/EPAS1/BACH1 regulatory axis, we performed a series of bioinformatic analyses. First, using the ENCORI database, we identified complementary binding sites between hsa-miR-138-5p and the 3′ untranslated region (3′UTR) of EPAS1, with the predicted seed sequence shown in Figure 7A.

Next, we assessed the co-expression relationship between EPAS1 and BACH1 in human whole blood using the GEPIA2 platform. Pearson correlation analysis revealed a significant positive correlation between their expression levels (r = 0.30, P = 2.6 × 10^−8^; Figure 7B), both EPAS1 and BACH1 were significantly upregulated in AMI samples in the GSE66360 dataset, suggesting that EPAS1 may transcriptionally activate the expression of BACH1 in the context of AMI.

To investigate whether EPAS1 could directly regulate BACH1 transcription, we retrieved the position weight matrix for EPAS1 (MA2325.1) from the JASPAR database (Figure 7C) and scanned the BACH1 promoter region (+2000 to −100 bp relative to the transcription start site) (Figure 7D) for putative binding motifs. Using a relative profile score threshold of ≥0.8, we identified two high-confidence EPAS1-binding sites within the BACH1 promoter (Figure 7E), supporting the possibility of direct transcriptional activation.

### 3.7 Diagnostic performance, tissue-specific expression profile and RT-qPCR validation of hub ferroptosis-related genes in AMI

**FIGURE 8:**
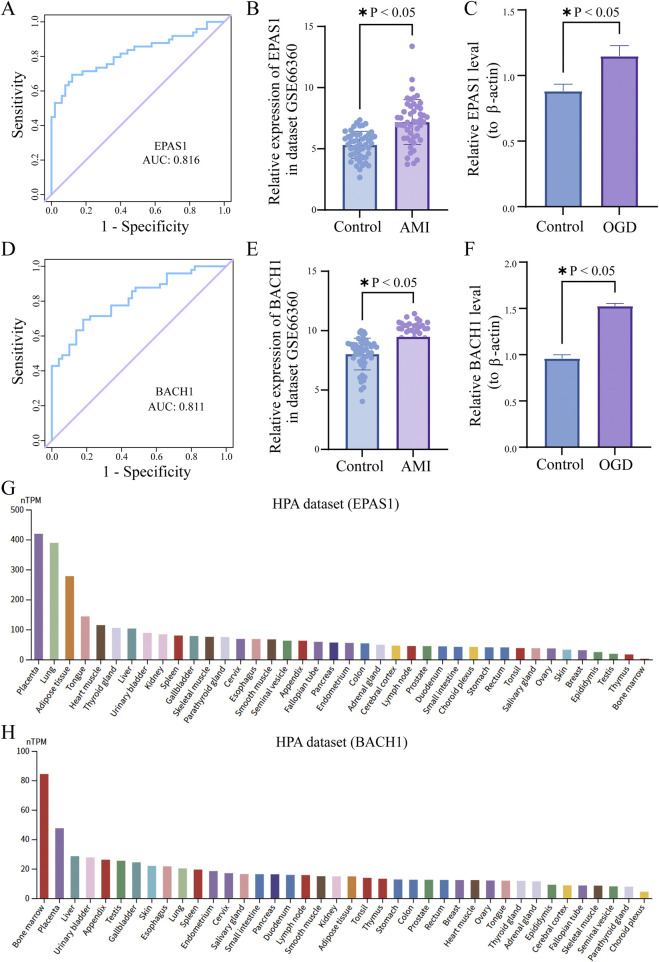
Diagnostic Performance and Differential Expression of EPAS1 and BACH1 in AMI, with Experimental Validation by RT-qPCR. **(A)** ROC curve analysis assessing the diagnostic value of EPAS1 in the GSE66360 cohort. **(B)** Bar plot showing EPAS1 expression levels in AMI patients versus controls across GEO datasets. ✱ indicates statistical significance (*P* < 0.05). All bar charts display data as mean ± standard deviation (SD) calculated from three independent biological replicates. **(C)** RT-qPCR analysis of EPAS1 expression in HUVECs. ✱ indicates statistical significance (*P* < 0.05). All bar charts display data as mean ± standard deviation (SD) calculated from three independent biological replicates. **(D)** ROC curve analysis assessing the diagnostic value of BACH1 in the GSE66360 cohort. **(E)** Bar plot showing BACH1 expression levels in AMI patients versus controls across GEO datasets. ✱ indicates statistical significance (*P* < 0.05). All bar charts display data as mean ± standard deviation (SD) calculated from three independent biological replicates. **(F)** RT-qPCR analysis of BACH1 expression in HUVECs. ✱ indicates statistical significance (*P* < 0.05). All bar charts display data as mean ± standard deviation (SD) calculated from three independent biological replicates. **(G,H)** Tissue expression profiles of EPAS1 and BACH1 in the Human Protein Atlas (HPA).

We evaluated the diagnostic potential of the key genes for distinguishing AMI patients from controls using ROC curve analysis based on the GSE66360 dataset. Both EPAS1 and BACH1 exhibited good diagnostic performance, with AUC values of 0.816 (95% CI: 0.730–0.903) and 0.811 (95% CI: 0.727–0.894), respectively (Figures 8A,D). Consistent with their upregulation in AMI, mRNA expression levels of EPAS1 and BACH1 were significantly higher in AMI samples compared to controls (Figures 8B,E). RT-qPCR was subsequently performed to verify the above findings. As expected, the mRNA levels of EPAS1 and BACH1 were markedly increased in OGD-treated HUVECs relative to controls, which was consistent with the transcriptome data (Figures 8C,F). To assess tissue distribution at the protein level, we analyzed data from The Human Protein Atlas. EPAS1 exhibited markedly elevated expression in specific tissues, including the placenta, lung, and adipose tissue, with expression levels exceeding 300 nTPM. By contrast, BACH1 showed relatively low expression across most tissues, generally around 20 nTPM; only the bone marrow and placenta presented moderately higher BACH1 expression, still below 100 nTPM (Figures 8G,H).

### 3.8 Functional association network and pathway enrichment of hub genes

**FIGURE 9:**
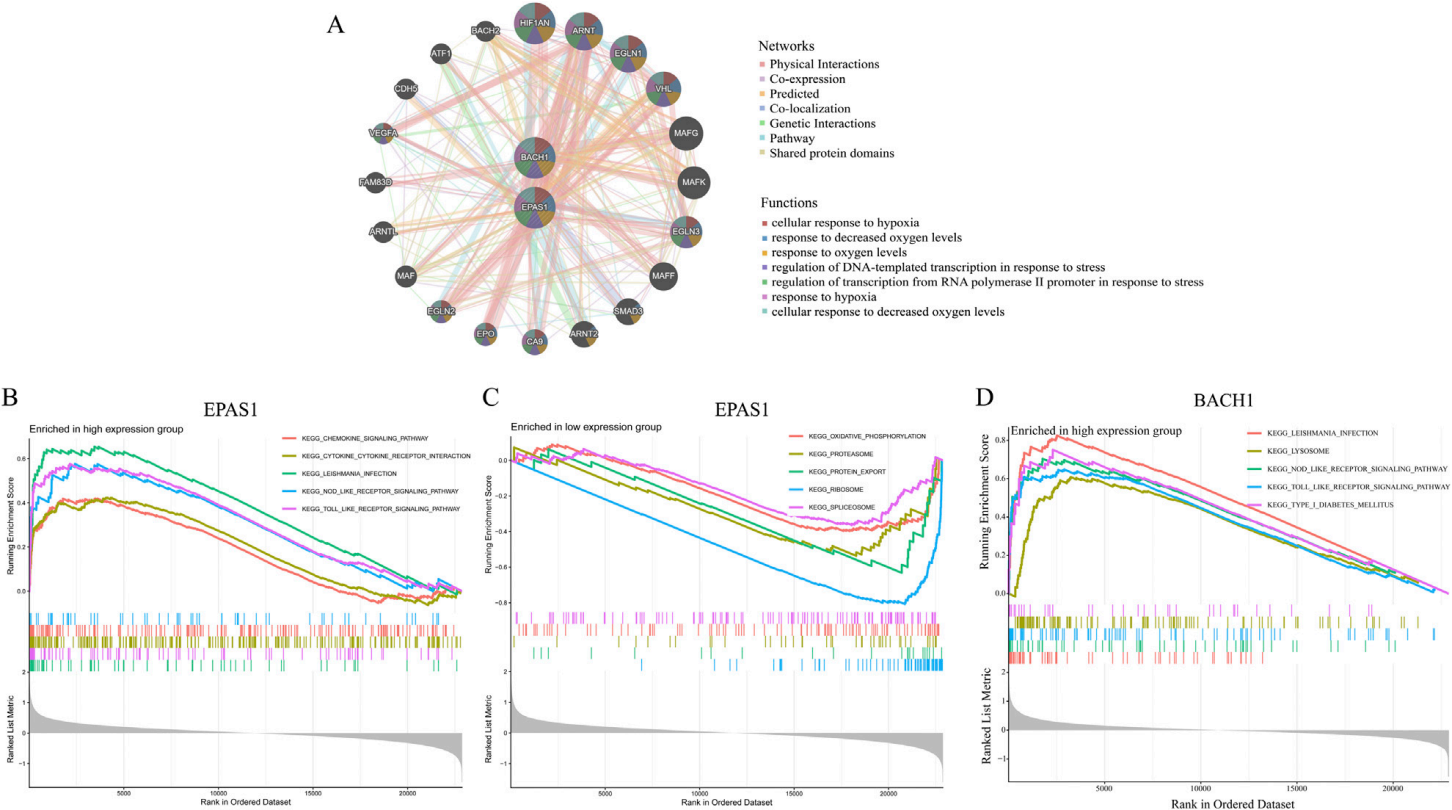
GeneMANIA and GSEA analysis results of EPAS1 and BACH1. **(A)** Result of GeneMANIA analysis. **(B-D)** Results of GSEA analysis.

GeneMANIA analysis revealed a dense functional association network centered on EPAS1 and BACH1, incorporating 20 top functionally related genes (Figure 9A). The network was primarily driven by co-expression and shared pathway annotations, with prominent connections to genes involved in hypoxia response, oxidative stress regulation, and iron metabolism. These associations suggest that EPAS1 and BACH1 are embedded within molecular modules critical for cellular responses to ischemic and oxidative stress, key processes in AMI pathogenesis.

GSEA was performed to explore signaling pathways associated with EPAS1 and BACH1 expression. A total of 99 AMI samples were stratified into high-expression and low-expression subgroups based on the “median expression value” of each gene. Approximately 50 samples were assigned to the high-expression group and 49 to the low-expression group. Genes were ranked according to the expression difference between the two subgroups. A “positive normalized enrichment score (NES)” indicated pathway enrichment in the high-expression subgroup, while a “negative NES” indicated enrichment in the low-expression subgroup. Pathways with adjusted P < 0.05 were considered statistically significant.

The results demonstrated a striking dichotomy in pathway activity dependent on EPAS1 expression levels. In samples with high EPAS1 expression, significant enrichment was observed for immune-inflammation-related pathways, including chemokine signaling pathway, cytokine–cytokine receptor interaction, leishmaniasis, NOD-like receptor signaling pathway, and Toll-like receptor signaling pathway (Figure 9B). Conversely, low EPAS1 expression was associated with enrichment of pathways involved in fundamental cellular processes, such as oxidative phosphorylation, proteasome, protein export, ribosome, and spliceosome (Figure 9C). Similarly, elevated BACH1 expression was significantly linked to the activation of several immune-lysosome-related pathways, including leishmaniasis, lysosome, NOD-like receptor signaling pathway, Toll-like receptor signaling pathway, and type I diabetes mellitus (Figure 9D). Notably, no KEGG pathways reached statistical significance (adjusted P < 0.05) in the GSEA analysis comparing samples with low BACH1 expression, suggesting a more limited or distinct functional scope under BACH1 downregulation. Collectively, these findings indicate that both EPAS1 and BACH1 are associated with the modulation of innate immune and inflammatory responses in AMI.

### 3.9 Gene-disease, gene-drug networks and PheWAS analysis of hub genes

**FIGURE 10:**
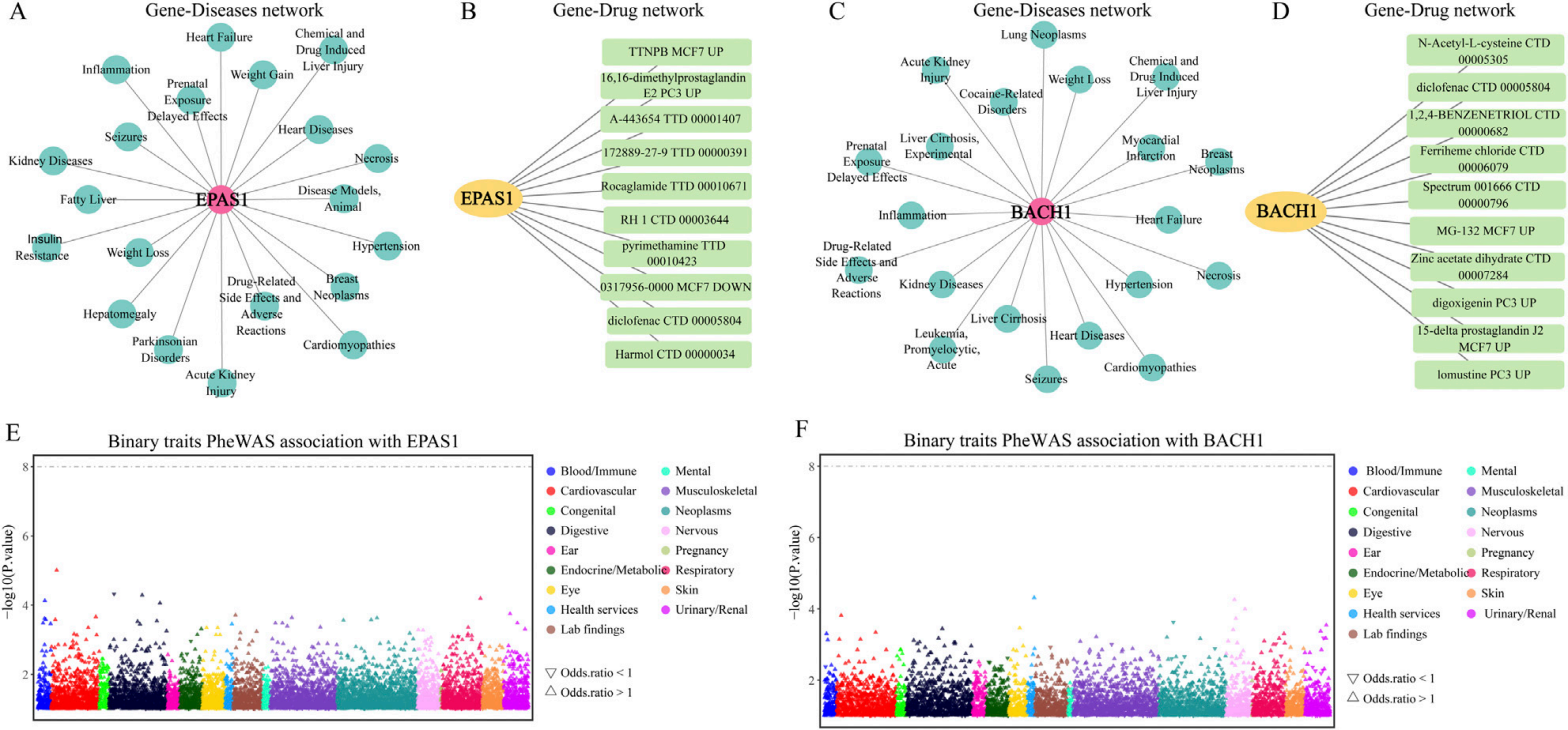
Gene-disease and gene-drug prediction and PheWAS. **(A-C)** Gene-disease networks predicted by the CTD database. **(B-D)** Gene-drug networks predicted by the MISGDB database. **(E)** Binary traits association with EPAS1. **(F)** Binary traits association with BACH1.

To assess the clinical and translational relevance of EPAS1 and BACH1, we performed integrative association analyses. Both genes showed strong evidence linking them to cardiovascular and inflammatory diseases, with top associations including cardiomyopathies, hypertension, and inflammation (Figures 10A,C, respectively).

Gene-drug interaction analysis using the MISGDB database identified multiple compounds with high-confidence associations for EPAS1 and BACH1. The top 10 drugs predicted to target each gene are listed in Tables 3, 4, respectively. Cytoscape network visualization further illustrated these interactions: EPAS1 was linked to compounds such as diclofenac, rocaglamide, and sorafenib (Figure 10B), while BACH1 showed strong associations with diclofenac, ferriheme chloride, and other candidate compounds (Figure 10D). It should be emphasized that the present study only screened candidate compounds with potential binding capacity to EPAS1 and BACH1 based on bioinformatic prediction. The regulatory effects of these compounds on the expression or activity of EPAS1 and BACH1 remain unclarified. Accordingly, these predicted compounds are merely provided as preliminary research candidates and are not suitable for direct clinical medication guidance.

**TABLE 3:** Candidate drugs of EPAS1 predicted using DSigDB.

Drug names	Pvalue	adj.pvalue	Genes
Diclofenac CTD 00005804	0.000	0.012	EPAS1
Rocaglamide TTD 00010671	0.002	0.012	EPAS1
172,889–27–9 TTD 00000391	0.002	0.012	EPAS1
A-443654 TTD 00001407	0.002	0.012	EPAS1
Harmol CTD 00000034	0.002	0.012	EPAS1
Pyrimethamine TTD 00010423	0.002	0.012	EPAS1
0317956–0000 MCF7 DOWN	0.002	0.012	EPAS1
16,16-Dimethylprostaglandin E2 PC3 UP	0.002	0.012	EPAS1
RH 1 CTD 00003644	0.002	0.012	EPAS1
TTNPB MCF7 UP	0.002	0.012	EPAS1

**TABLE 4:** Candidate drugs of BACH1 predicted using DSigDB.

Drug names	Pvalue	adj.pvalue	Genes
Diclofenac CTD 00005804	0.000	0.012	BACH1
Ferriheme chloride CTD 00006079	0.005	0.012	BACH1
Spectrum 001,666 CTD 00000796	0.009	0.017	BACH1
1,2,4-BENZENETRIOL CTD 00000682	0.010	0.017	BACH1
15-Delta prostaglandin J2 MCF7 UP	0.012	0.020	BACH1
Lomustine PC3 UP	0.015	0.022	BACH1
Zinc acetate dihydrate CTD 00007284	0.015	0.022	BACH1
MG-132 MCF7 UP	0.027	0.035	BACH1
Digoxigenin PC3 UP	0.030	0.038	BACH1
N-Acetyl-L-cysteine CTD 00005305	0.032	0.039	BACH1

To evaluate potential off-target phenotypic effects, we performed PheWAS analysis using the AstraZeneca PheWAS Portal. Visualization of association results for EPAS1 and BACH1 is shown in Figures 10E,F, respectively. Across a wide range of tested phenotypes, including mental, nervous, skin, and digestive traits, no associations reached the genome-wide significance threshold (P < 5 × 10^−8^). These findings reveal no genome-wide significant adverse phenotypic associations linked to EPAS1 and BACH1 genetic variants. However, these genetic-level neutral signals cannot fully guarantee pharmacological safety under ischemic stress; the two genes merely represent candidate molecules requiring further safety and efficacy verification in preclinical models.

## 4 Discussion

This study integrates transcriptomics analysis, SMR, and colocalization methods, and for the first time identifies EPAS1 as a key transcription factor closely associated with ferroptosis and having a causal role in AMI. Furthermore, a miRNA-EPAS1-mRNA regulatory network was constructed, and based on the endothelial cell context, a new ferroptosis regulatory axis was proposed: hsa-miR-138-5p/EPAS1/BACH1. This axis couples the post-transcriptional inhibitory effects of miRNAs with the regulation of downstream target genes by transcription factors, revealing the potential molecular mechanisms of the ferroptosis program in endothelial cells.

EPAS1, also known as HIF-2α, is a hypoxia-inducible bHLH-PAS transcription factor that dimerizes with HIF-1β to bind hypoxia-response elements (HREs) and regulate adaptive processes including erythropoiesis, angiogenesis, iron metabolism, and oxidative stress responses (Lu et al., 2024). EPAS1 has been shown to protect against early atherosclerosis by regulating endothelial fatty acid uptake at sites of disturbed blood flow. Its expression in endothelial cells is controlled by both hemodynamic shear stress and metabolic cues, with reduced EPAS1 contributing to lesion initiation (Pirri et al., 2024; Sluimer, 2024). In contrast to these protective roles in chronic vascular disease, our study identifies a detrimental function of EPAS1 in AMI, where it promotes ferroptosis and cardiac injury.

Our findings are consistent with recent bioinformatic studies implicating EPAS1 as a ferroptosis-associated gene that exacerbates AMI (Huang et al., 2024). However, these prior analyses were largely based on heart tissue transcriptomics and lacked causal inference or cell-type resolution. In contrast, we leveraged HUVECs and an endothelial cell-derived dataset to investigate EPAS1 expression. Both *in vitro* experiments and bioinformatics analysis demonstrated significant upregulation of EPAS1 under AMI conditions. More importantly, SMR and colocalization deliver suggestive genetic evidence linking elevated EPAS1 expression to higher AMI risk. Our genetic analyses support EPAS1 as a potential susceptibility locus rather than a confirmed direct pathogenic driver; its pro-ferroptosis effect in endothelial cells remains to be fully validated via functional experiments.

This study offers three major contributions. First, SMR and colocalization analyses yield suggestive genetic associations between EPAS1 expression and AMI susceptibility, indicating EPAS1 as a candidate risk-related gene rather than a verified disease driver. Second, we characterize a novel endothelial-enriched regulatory axis hsa-miR-138-5p/EPAS1/BACH1 that mediates ferroptosis under ischemic endothelial injury. Third, *in silico* drug screening identifies candidate compounds potentially interacting with EPAS1 and BACH1, while PheWAS detects no genome-wide harmful phenotypic correlations for variants in these genes. Nevertheless, such genetic safety signals cannot replace pharmacological verification, and this regulatory axis only serves as a research candidate for follow-up preclinical testing, not a validated therapeutic target for AMI.

Several limitations of this study should be acknowledged. First, as the GTEx database does not currently provide eQTL data for endothelial cells, whole blood samples were used as the data source for SMR analysis in this study. However, both an endothelial cell-derived dataset and OGD-treated HUVECs consistently demonstrated significant EPAS1 upregulation, confirming its elevation in AMI-related endothelial injury. Although RT-qPCR validated the upregulated mRNA expression of EPAS1 and BACH1 in OGD-induced endothelial injury consistent with transcriptome datasets, this study only confirmed transcriptional expression changes at the mRNA level. We acknowledge two key limitations of the current *in vitro* validation: (1) No Western blot was performed to detect EPAS1 and BACH1 protein abundance, so post-transcriptional or translational regulatory alterations cannot be excluded; (2) Direct ferroptosis functional verification experiments including lipid ROS measurement, intracellular iron accumulation detection and GPX4 activity assay were not conducted. Therefore, the ferroptosis-promoting biological effects of EPAS1/BACH1 in endothelial cells remain preliminary and require further functional validation in subsequent experiments. Our diagnostic discrimination analysis was only performed on CEC transcriptome data from a single AMI discovery cohort (GSE66360). GSE71226 cannot provide independent validation for AMI-specific biomarkers due to mixed coronary heart disease phenotypes. Although we lack an extra clinical AMI transcriptomic dataset for external validation, *in vitro* OGD-HUVEC experiments verified that the upregulation of EPAS1 and BACH1 under endothelial ischemic injury matched the transcriptional signature in clinical AMI samples. Future verification based on large independent pure AMI patient cohorts with complete clinical metadata is still required to further confirm the discriminatory value of EPAS1 and BACH1. Second, the proposed regulatory axis involving hsa-miR-138-5p, EPAS1, and BACH1 was primarily inferred from bioinformatic predictions and expression correlations, and lacks direct experimental support. Subsequently, we will use HUVECs as the research model to carry out *in vitro* functional experiments. With the help of dual-luciferase reporter genes, miRNA overexpression/silencing, and Western blot techniques, we will systematically verify the targeting regulatory effect of miR-138-5p on EPAS1 in a homologous cell system, compensating for the limitation of inconsistent sample sources in this study and improving the molecular mechanism evidence chain. Third, although we rigorously filtered genetic instruments using HEIDI and LD clumping to minimize heterogeneity and linkage disequilibrium, residual horizontal pleiotropy cannot be entirely ruled out in SMR-based causal inference. Fourth, the biomarker-related analyses in this study have obvious limitations in clinical translational verification. All transcriptome data were derived from the public GEO dataset GSE66360 with purified circulating endothelial cells, and we could not obtain additional independent AMI cohorts with matched endothelial transcriptomic profiles and complete clinical covariate data (including age, gender, hypertension, diabetes, blood lipid levels, time from symptom onset to reperfusion, cardiac function classification and other confounding factors). Therefore, multivariable regression models correcting clinical confounders and cross-cohort external validation were not achievable in the current work. The ROC curves only reflected intra-cohort differential expression discrimination of EPAS1 and BACH1 in circulating endothelial cells, and cannot support their application as routine clinical diagnostic markers for AMI. Despite this limitation, circulating endothelial cells are specifically shed from damaged coronary endothelium during AMI, and the stable upregulation of EPAS1 and BACH1 in this endothelial-specific cell population still endows the two genes with value as exploratory markers for evaluating endothelial ischemic injury in basic myocardial infarction research and *in vitro* OGD endothelial models. In future prospective clinical studies, we will recruit independent AMI patient cohorts, collect complete clinical metadata, construct multivariable prediction models adjusted for multiple cardiovascular confounders, and perform cross-cohort validation to further verify the clinical translational potential of EPAS1 and BACH1. Finally, while our drug-repurposing analysis identified promising candidates, future studies will prioritize preclinical evaluation of these candidates in relevant AMI models, assessing their impact on ferroptosis and cardiac function. Specifically, our *in silico* drug repurposing predictions identifying diclofenac and other compounds targeting EPAS1/BACH1 cannot be directly extrapolated to clinical cardiovascular outcomes in AMI patients, with several critical translational hurdles remaining unresolved. First, database-based gene-drug associations only predict potential molecular binding capacity without confirming endothelial-specific drug enrichment within the hypoxic myocardial ischemic microenvironment. Second, computational screening cannot evaluate systemic hemodynamic side effects of candidate agents. As a typical representative, diclofenac may induce fluid retention and elevated blood pressure in clinical populations, which could counteract its potential anti-ferroptosis cardioprotective effects mediated by EPAS1/BACH1 inhibition. Third, while our PheWAS analysis detected no genome-wide significant adverse phenotypic associations linked to EPAS1 and BACH1 genetic variants, these genetic-level neutral signals reflect long-term baseline genetic susceptibility rather than acute pharmacological responses under ischemic stress, so they cannot fully guarantee the safety of short-term drug intervention during AMI. To bridge these predictive omics findings with experimental evidence, future layered preclinical validations are required: (1) *In vitro* OGD-HUVEC models will be used to screen effective drug concentrations that suppress EPAS1/BACH1 and reduce endothelial ferroptosis; (2) Mouse myocardial ischemia/reperfusion models will be adopted to evaluate compound efficacy in reducing infarct size while monitoring systemic hemodynamic parameters to balance therapeutic benefits and cardiovascular risks; (3) Retrospective clinical cohort analysis will be performed to explore the correlation between diclofenac exposure and AMI-related adverse events, further consolidating the translational interpretation of our PheWAS results.

## 5 Conclusion

In conclusion, our multi-omics integrative analyses identify EPAS1 as an endothelial-enriched transcription factor with suggestive genetic links to AMI susceptibility and characterize a ferroptosis-associated hsa-miR-138-5p/EPAS1/BACH1 regulatory cascade preferentially expressed in endothelial cells. This work offers mechanistic clues regarding iron-dependent cell death during myocardial ischemia. In silico drug prediction and PheWAS analysis supply preliminary genetic safety hints for EPAS1 and BACH1, though further *in vitro* and *in vivo* functional validation is mandatory to judge their druggable potential. Collectively, this study lays a bioinformatic foundation for future preclinical research exploring endothelial ferroptosis in AMI.

## Data Availability

The public datasets analyzed in this study were obtained from the Gene Expression Omnibus (GEO) database under accession numbers GSE66360, GSE71226, and GSE148153. For SMR analysis, exposure data were derived from the GTEx project, and outcome GWAS summary statistics were accessed from IEU OPEN GWAS. The original contributions presented in the study are included in the article/Supplementary Material, further inquiries can be directed to the corresponding author.
